# Control of the Actin Cytoskeleton Within Apical and Subapical Regions of Pollen Tubes

**DOI:** 10.3389/fcell.2020.614821

**Published:** 2020-12-03

**Authors:** Yanan Xu, Shanjin Huang

**Affiliations:** Center for Plant Biology, School of Life Sciences, Tsinghua University, Beijing, China

**Keywords:** pollen tube growth, cytoplasmic streaming, actin dynamics, apical actin structure, actin-binding proteins, formin, ADF, villin

## Abstract

In flowering plants, sexual reproduction involves a double fertilization event, which is facilitated by the delivery of two non-motile sperm cells to the ovule by the pollen tube. Pollen tube growth occurs exclusively at the tip and is extremely rapid. It strictly depends on an intact actin cytoskeleton, and is therefore an excellent model for uncovering the molecular mechanisms underlying dynamic actin cytoskeleton remodeling. There has been a long-term debate about the organization and dynamics of actin filaments within the apical and subapical regions of pollen tube tips. By combining state-of-the-art live-cell imaging with the usage of mutants which lack different actin-binding proteins, our understanding of the origin, spatial organization, dynamics and regulation of actin filaments within the pollen tube tip has greatly improved. In this review article, we will summarize the progress made in this area.

## Introduction

In flowering plants (angiosperms), seed formation requires two fertilization events. The male germ unit, called the male gametophyte, is contained within pollen grains and comprises a vegetative cell and two sperm cells that have already lost their motility (Kaul et al., 2000; Dresselhaus et al., 2016; Higashiyama, 2018). The process of double fertilization begins when pollen grains land on and adhere to the surface of the stigma. Following hydration of the pollen grain, the vegetative cell generates a pollen tube (Chapman and Goring, 2010). Pollen tubes then grow through the transmitting tissue of the style and serve as an active vehicle to transport the two immotile sperm cells into the ovule under the attraction of various female molecules (Higashiyama and Takeuchi, 2015; Zhang et al., 2017; Johnson et al., 2019). Therefore, pollen tube growth represents a critical stage during the extended journey that is required for double fertilization in flowering plants.

Similar to the filamentous protonemata of mosses and the root hairs of high plants, pollen tubes are tip-growing cells, with growth strictly occurring within the apical region (Rounds and Bezanilla, 2013). Pollen tubes grow rapidly both *in vivo* and *in vitro*. For instance, the growth rate of lily (*Lilium longiflorum* and *Lilium formosanum*) pollen tubes can reach up to 12–18 μm min^–1^ (Hepler et al., 2001). Although *Arabidopsis thaliana* pollen tubes and *Nicotiana tabacum* pollen tubes grow comparatively slowly when compared to lily pollen tubes, their growth rate can reach up to 2 and 1.5–6 μm min^–1^, respectively (Cheung and Wu, 2008). The rapidity of pollen tube growth greatly shortens the time required for the delivery of sperm cells to the ovules, thus favoring fertilization. Plant biologists have been fascinated by this remarkable growth phenomenon. The rapid growth of pollen tubes requires the availability of a huge amount of materials for plasma membrane expansion and cell wall synthesis within the pollen tube growth region. In line with this, pollen tubes harbor an active intracellular transport system to enable the efficient delivery of materials to the growth region, which subsequently coordinate with tightly regulated endocytotic and exocytotic events to support pollen tube tip growth (Rounds and Bezanilla, 2013). The actin cytoskeleton plays an essential role in driving the growth and morphogenesis of pollen tubes by choreographing endo- and exocytotic vesicle traffic (Taylor and Hepler, 1997; Cole and Fowler, 2006; Cheung and Wu, 2008; Yang, 2008; Qin and Yang, 2011; Guan et al., 2013). As such, the role and mechanism of action of the actin cytoskeleton in the regulation of polarized pollen tube growth have been subjected to intensive scrutiny in the past few decades. Careful examination of the organization of actin filaments in living and fixed pollen tubes has provided significant insights into the spatial organization of actin filaments in pollen tubes (for reviews see Hepler et al., 2001; Vidali and Hepler, 2001; Samaj et al., 2006; Ren and Xiang, 2007; Chen et al., 2009; Staiger et al., 2010; Cai et al., 2015; Fu, 2015; Qu et al., 2015; Stephan, 2017). In this review, we will describe our current understanding of the organization, dynamics and regulation of the actin cytoskeleton in pollen tubes, with the focus on the apical and subapical regions.

## The Actin Cytoskeleton in Pollen Tubes

**FIGURE 1:**
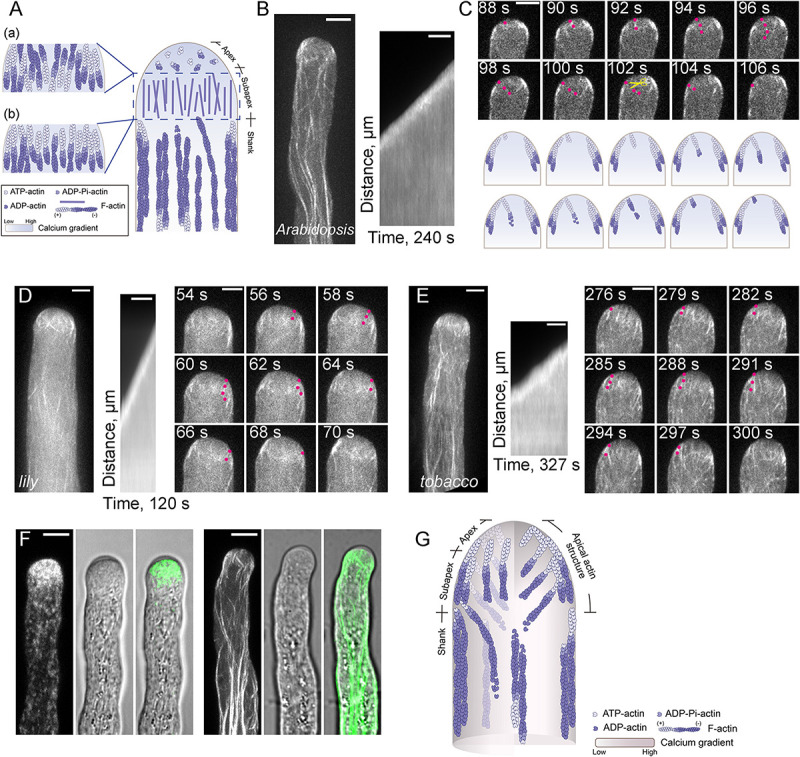
Actin Filaments are Continuously Polymerized from the Plasma Membrane within the Apical and Subapical Regions of the Pollen Tube. **(A)** Schematic diagram depicting our previous understanding of the spatial distribution of actin filaments in the pollen tube. This model refers to the models shown in previous review articles with slight modifications (Vidali and Hepler, 2001; Lovy-Wheeler et al., 2005; Ren and Xiang, 2007; Cheung and Wu, 2008; Yang, 2008; Cai and Cresti, 2009; Qin and Yang, 2011; Guan et al., 2013; Rounds and Bezanilla, 2013; Cai et al., 2015; Fu, 2015; Bascom et al., 2018). Specifically, actin filaments are arrayed into longitudinally oriented actin bundles in the shank and in the actin fringe structure at the subapex. In terms of the polarity of actin filaments within the actin fringe, the models in (a,b) were drawn with reference to Ren and Xiang (2007); Cheung and Wu (2008), and Qin and Yang (2011), respectively. By comparison, actin filaments at the apex are short, less abundant and disorganized. **(B)** Actin filaments are polymerized from the plasma membrane in an *Arabidopsis* pollen tube tip. The right panel shows the kymograph analysis of apical actin filaments decorated with Lifeact-eGFP in the growing wild-type (WT) *Arabidopsis* pollen tube shown in the left panel. Scale bar = 5 μm. **(C)** Time-lapse images of apical actin filaments in the pollen tube shown in **(B)**. Red dots indicate an actin filament that is polymerized from the plasma membrane, then grows into the inner region of the pollen tube. The yellow scissors indicate a severing event of the same actin filament. Scale bar = 5 μm. The lower panel shows a schematic depiction of the events in the upper panel. **(D,E)** Actin filaments polymerized from the plasma membrane at the tip of a growing lily **(D)** and tobacco **(E)** pollen tube. In each figure, the left panel shows the Z-projection image of actin filaments in the pollen tube. The middle panel shows kymograph analysis of actin filaments growing from the plasma membrane at the pollen tube tip, and the right panel shows some time-lapse images of actin filaments in the growing pollen tube shown in the left panel. Red dots indicate an actin filament that was polymerized from the plasma membrane, then grew into the inner region of the pollen tube. Scale bar = 5 μm. **(F)** Visualization of RabA4b-positive transport vesicles (left panel) and actin filaments (right panel) in WT *Arabidopsis* pollen tubes. Transport vesicles accumulate within the region corresponding to the clear zone at the pollen tube tip (left panel). Actin filaments at the base of the clear zone, which polymerize from the plasma membrane, correspond to the actin fringe at the subapex shown in **(A)**. Scale bar = 5 μm. **(G)** Schematic depiction of our current view of the organization of actin filaments in the *Arabidopsis* pollen tube. Similar to the model shown in **(A)**, actin filaments are organized into actin bundles oriented longitudinally in the shank region. Within the apical and subapical regions of the pollen tube, actin filaments are polymerized from the plasma membrane. These filaments can be viewed as a whole and defined as the “apical actin structure.” Membrane-originated actin filaments within this “apical actin structure” assume a distinct spatial distribution, with some cortical actin filaments forming thick actin bundles, while some inner actin filaments are comparatively fine and extend toward the inner region of the cytoplasm.

Experimental treatments with actin-based pharmacological agents showed that the actin cytoskeleton is absolutely required for pollen germination and pollen tube growth (Franke et al., 1972; Herth et al., 1972; Mascarenhas and Lafountain, 1972; Speranza and Calzoni, 1989; Heslopharrison and Heslopharrison, 1991; Mascarenhas, 1993; Gibbon et al., 1999; Vidali et al., 2001). As the building block of the actin cytoskeleton, actin is a very abundant protein in pollen, accounting for about 2–20% of the total soluble protein in pollen grains (Liu and Yen, 1992; Ren et al., 1997; Vidali and Hepler, 1997; Gibbon et al., 1999; Snowman et al., 2002). Therefore, plant scientists have used pollen as the starting material to isolate polymerization-competent plant actin (Liu and Yen, 1992; Ren et al., 1997). Different methods have been used to determine the cellular concentration of actin monomers in pollen from maize (Gibbon et al., 1999), poppy (Snowman et al., 2002), lily (Vidali and Hepler, 1997), and *Arabidopsis* (Jiang et al., 2019). These investigations showed that the total actin concentration can reach up to about 200 μM in pollen. In fact, there are five reproductive actin isovariants (*ACT1*, *ACT3*, *ACT4*, *ACT11*, and *ACT12*) expressed in *Arabidopsis* pollen, and simultaneous silence of *ACT1*, *ACT3*, *ACT4*, and *ACT12* by RNA interference (RNAi) causes obvious reproductive defects (Pawloski et al., 2006). The direct evidence for the involvement of actin in regulating pollen germination and pollen tube growth came from the analysis of the mutant lacking *ACT11*, showing that pollen germination was inhibited (Chang and Huang, 2015). Surprisingly, loss of function of *ACT11* upregulates pollen tube growth (Chang and Huang, 2015), which is presumably due to the increase in actin dynamics in pollen tubes. Given that the local concentration of actin monomers directly impacts their assembly and disassembly, researchers in this field have tried to reveal the intracellular localization of actin monomers in pollen tubes. They showed that there exists a tip-focused gradient of monomeric G-actin in pollen tubes (Li et al., 2001; Cardenas et al., 2005). One interesting study showed that actin monomers actually distribute uniformly within the cytoplasm of *Arabidopsis* pollen tubes and are rapidly redistributed via cytoplasmic streaming (Chang et al., 2017), which suggests that actin monomers are readily available to assemble within the pollen tube. Given that most actin-based functions are carried out by the filamentous form (F-actin), plant scientists have tried different methods to uncover the organization of actin filaments in pollen tubes. These approaches include labeling actin filaments with fluorescently-tagged phalloidin or immunostaining with an anti-actin antibody in fixed pollen tubes (Tang et al., 1989; Gibbon et al., 1999; Geitmann et al., 2000; Lovy-Wheeler et al., 2005; Thomas et al., 2006; Wilsen et al., 2006; Ye et al., 2009; Wu et al., 2010; Zhang et al., 2010; Qu et al., 2020), or using actin markers including GFP-mTalin or YFP-mTalin, GFP-ABD2, GFP-ADF, GFP-LIM, and Lifeact-GFP or YFP-Lifeact (Kost et al., 1998; Fu et al., 2001; Wilsen et al., 2006; Cheung et al., 2008; Vidali et al., 2009; Zhang et al., 2009; Zhang et al., 2010; Qu et al., 2013; Stephan et al., 2014) to decorate actin filaments in living pollen tubes. These investigations have resulted in a consensus view that actin filaments are arranged into longitudinally aligned bundles in the shank region of pollen tubes (Figure 1A). Shank actin filaments are important for a transport mechanism in angiosperm pollen tubes called reverse-fountain cytoplasmic streaming. The flow of cytoplasm is generated by the movement of barbed-end directed myosin motors along the shank-localized actin filaments. In the cortex of the pollen tube, cytoplasm flows toward the tip, while in the middle of the pollen tube, it flows back toward the bottom. Based on the determination of the polarity of shank-localized actin bundles in root hairs (Tominaga et al., 2000), which also generate reverse-fountain cytoplasmic streaming, cortical actin bundles and inner actin bundles likely have their barbed ends facing toward the tip and base of pollen tubes, respectively. Indeed, this has been verified by visualizing actin filaments decorated with myosin II subfragment 1 in pollen tubes by electron microscopy (Lenartowska and Michalska, 2008). Both cortical and inner actin bundles terminate at the subapex of pollen tubes. By comparison, determining the organization of the actin cytoskeleton within the apical and subapical regions has been problematic, as different configurations have been reported in pollen tubes from different species using different methods. In the following sections, we will focus on describing our understanding of the organization, dynamics and regulation of the actin cytoskeleton within the apical and subapical regions of pollen tubes.

## The Origin and Spatial Organization of Actin Filaments Within the Apical and Subapical Regions of Pollen Tubes

Previous studies suggested that pollen tube growth is more sensitive to the treatment of actin destabilizing reagents than cytoplasmic streaming (Gibbon et al., 1999; Vidali et al., 2001), which suggests that the actin cytoskeleton within the pollen tube growth region is highly dynamic. This is also the reason why the actin cytoskeleton within the apical and subapical regions cannot be fixed instantly, thus preventing us from reaching a consensus view about the organization of the actin cytoskeleton within the pollen tube growth region. In the past, efforts have been made to describe the organization of the actin cytoskeleton within the apical and subapical regions separately. Although there is some argument about whether actin filaments exist within the apical region of pollen tubes, researchers in the field believe that the apical region does contain actin filaments, but they are short, less abundant and randomly distributed (Yang, 2008; Staiger et al., 2010). Different organizational patterns of subapical actin filaments within pollen tubes have been revealed by different actin labeling approaches and, as such, different names have been provided to describe the organization of subapical actin filaments in pollen tubes from different species. These names include actin collar (Gibbon et al., 1999; Qu et al., 2013), actin fringe (Lovy-Wheeler et al., 2005; Dong et al., 2012; Rounds et al., 2014), actin ring or actin mesh (Kost et al., 1998; Chen et al., 2002). The variation in the subapical actin structure might be due to the employment of different actin labeling approaches or to true differences among pollen tubes from different species. Different schematic models had been generated to describe the organization of actin filaments within the apical and subapical regions of pollen tubes. One typical schematic model, presented in Figure 1A, shows that actin filaments are arranged into an actin fringe structure at the subapex, and are shorter, less abundant and more disorganized at the extreme apex. However, the polarity of actin filaments within the actin fringe remains the subject of debate. Different models are presented in the literature in terms of the polarity of actin filaments within the actin fringe structure. One model showed that actin filaments at the cortex and in the inner region within the actin fringe have their barbed ends facing toward the tip and base of the pollen tube, respectively (Figure 1Aa; Ren and Xiang, 2007; Cheung and Wu, 2008). Another model showed that actin filaments within the actin fringe have their barbed ends facing toward the tip of the pollen tube (Figure 1Ab; Qin and Yang, 2011). Therefore, the origin and exact organization of subapical and apical actin filaments are unclear.

In this regard, live-cell imaging of Lifeact-GFP-decorated actin filaments in growing wild-type pollen tubes and in mutant *Arabidopsis* pollen tubes with loss of function of specific actin-binding proteins (ABPs) has revolutionized our view about the origin, polymerization and organization of actin filaments within the apical and subapical regions of pollen tubes. Specifically, this approach has shown that actin filaments are continuously polymerized from the plasma membrane at the growing *Arabidopsis* pollen tube tips (Figures 1B,C; Qu et al., 2013). A similar phenomenon was also noticed in lily and tobacco pollen tubes (Figures 1D,E; Vidali et al., 2009; Rounds et al., 2014), which suggests that the polymerization of actin filaments from the apical plasma membrane is a common design in angiosperm pollen tubes. In support of this notion, loss of function of class I formins, which are important actin nucleating factors in pollen tubes, impairs the polymerization of actin filaments from the plasma membrane at pollen tube tips (Cheung et al., 2010; Lan et al., 2018). In line with this finding, loss of function of profilins, the functional partners of formins, impaired the polymerization of actin filaments from the plasma membrane at the extreme apex of pollen tubes (Liu et al., 2015). Simultaneous visualization of actin filaments, the clear zone (which corresponds to the previously defined actin fringe) and transport vesicles showed that the actin structure at the base of the clear zone (Lovy-Wheeler et al., 2005, 2006) is made up of actin filaments polymerized from the plasma membrane (Figure 1F; Qu et al., 2017). These findings allow us to propose a schematic model describing the spatial organization of apical and subapical actin filaments in the pollen tube (Figure 1G). In this model, actin filaments within both the apical and subapical regions of pollen tubes are generated from the plasma membrane, and the actin filaments within the two regions can be viewed as a whole, which is defined as the “apical actin structure” (Figure 1G; Qu et al., 2017). Consequently, actin filaments can be viewed as forming two notable structures in pollen tubes: the shank-localized longitudinal actin bundles and the “apical actin structure” (Qu et al., 2017).

## Molecular Mechanism Underlying the Regulation of Actin Polymerization From the Plasma Membrane in Pollen Tubes

Live-cell imaging of the dynamics of actin filaments revealed that actin polymerization continuously occurs from the plasma membrane at pollen tube tips, and this polymerization is required for and concurrent with pollen tube growth (Qu et al., 2017). How apical actin polymerization is regulated during pollen tube growth is an interesting question. Actin polymerization is dictated by specific actin nucleation factors, and Arp2/3 complex and formins are two major types of actin nucleation factors that have been characterized in plants (Blanchoin and Staiger, 2010). Both Arp2/3 complex and formins have received widespread attention in the context of plasma membrane-originated actin polymerization. The role of Arp2/3 complex in regulating the morphogenesis of trichome and epidermal pavement cells has been studied extensively (Le et al., 2003; Li et al., 2003; Mathur et al., 2003a,b; El-Din El-Assal et al., 2004), but the role of Arp2/3 complex in regulating actin polymerization in pollen is not clear. The tips of wild-type pollen tubes do not contain dense branched F-actin networks (Qu et al., 2015), and loss of function of Arp2/3 complex does not affect fertility in *Arabidopsis* (Szymanski, 2005), which indicates that the Arp2/3 complex is not essential for pollen tube growth. Therefore, there is no direct evidence that Arp2/3 complex is involved in the regulation of actin polymerization in pollen tubes. Formin proteins contain the characteristic formin homology (FH) domains, FH1 and FH2, and are able to nucleate actin assembly from actin monomers or actin-profilin complexes (Kovar, 2006; Goode and Eck, 2007). Based on the sequence of their FH2 domains, plant formins are divided into three classes, class I, class II, and class III (Cvrckova et al., 2004). Only two class III formins have been identified, and they are found in land plants that contain flagellate sperm (Grunt et al., 2008; van Gisbergen and Bezanilla, 2013). Class I and class II formins are common in plants. Most of the class I formins contain a transmembrane (TM) domain at their N-terminus, which enables them to target to the plasma membrane or endomembrane systems. The N-terminus of class II formins is quite variable. Some of them have a phosphatase and tensin homolog (PTEN)-like domain at their N-terminus (Blanchoin and Staiger, 2010). Considering that actin is buffered by an almost equimolar amount of profilin (Vidali and Hepler, 1997; Gibbon et al., 1999; Snowman et al., 2002; Jiang et al., 2019), and actin-profilin complexes are favored by formins rather than the Arp2/3 complex (Rotty et al., 2015; Suarez et al., 2015), it is easy to imagine the important role of formins in controlling actin polymerization in pollen. Accordingly, it was shown that treatment with the formin inhibitor SMIFH2 (Rizvi et al., 2009), which inhibits plant formins *in vitro* (Cao et al., 2016), impairs actin polymerization from the plasma membrane at pollen tube tips (Qu et al., 2017). As actin polymerization continuously occurs from the plasma membrane at the pollen tube tip (Qu et al., 2013), the TM-containing class I formins are particularly relevant. Indeed, two class I formins, *Arabidopsis* formin 3 (AtFH3), and AtFH5, have been shown to nucleate actin assembly from actin monomers or actin bound to profilin (Ingouff et al., 2005; Ye et al., 2009), and are redundantly required for actin polymerization from the plasma membrane in pollen tubes (Lan et al., 2018). Accordingly, reducing the expression of *Nicotiana tabacum* homolog of *AtFH5*, *NtFH5*, in tobacco pollen impairs the actin polymerization from the plasma membrane (Cheung et al., 2010). In line with this finding, overexpression of *Arabidopsis* formin 1 induces the formation of supernumerary actin cables from the plasma membrane and causes membrane deformation (Cheung and Wu, 2004). The importance of class I formins in regulating actin polymerization at pollen tube tips is also supported by the finding that the pollen-specific *Lilium longiflorum Formin 1* (*LlFH1*) controls the construction of the actin fringe in pollen tubes (Li et al., 2017). These findings together suggest that class I formins play important roles in controlling actin polymerization within the apical and subapical regions of pollen tubes.

Functional characterization of *Arabidopsis* profilins in pollen also provides evidence that formin is a major player in controlling actin polymerization at the tip of pollen tubes. Profilin is a low molecular weight protein, ranging from 12 to 15 kDa, and it can bind to G-actin to form high affinity 1:1 profilin-actin complexes (Carlsson et al., 1977; Vidali and Hepler, 1997). It was shown that profilin has a dual role in regulating actin dynamics. When the barbed ends of actin filaments are capped, profilin acts as a simple actin monomer sequestering protein to promote actin depolymerization (Huang et al., 2004). In support of this notion, it was shown that microinjection of profilin into *Tradescantia blossfeldiana* stamen hair cells causes the disappearance of transvacuole strands and displacement of nuclei (Staiger et al., 1994). However, when the barbed ends of actin filaments are free, actin-profilin complexes can add onto the barbed ends to elongate actin filaments and thus promote actin polymerization (Pantaloni and Carlier, 1993). Evidence for such a functional role of profilin was strengthened by the finding that the presence of formin can facilitate the addition of actin-profilin complexes onto the barbed ends of actin filaments to accelerate their elongation (Romero et al., 2004; Kovar et al., 2006). Within this framework, formin facilitates the addition of actin-profilin complexes onto the barbed end of actin filaments through its proline-rich FH1 domain. Consistent with this, it was shown that the function of profilin depends on its interaction with proline-rich motifs (Gibbon et al., 1998). Based on the fact that actin binds to profilin with high affinity (Gibbon et al., 1997; Kovar et al., 2000) and they exist in roughly equimolar amounts in pollen (Vidali and Hepler, 1997; Gibbon et al., 1999; Snowman et al., 2002; Jiang et al., 2019), it was predicted that actin mainly exists in the form of actin-profilin complexes in pollen (Staiger and Blanchoin, 2006; Chen et al., 2009). In support of the role of profilin in promoting actin polymerization, it was shown that loss of function of profilins impairs actin polymerization from the plasma membrane at the tip of *Arabidopsis* pollen tubes (Liu et al., 2015). Importantly, it was shown that the mutant PRF5_*Y6A*_, which is defective in binding to PLP but retains normal G-actin binding activity, has impaired function in actin polymerization at pollen tube tips (Liu et al., 2015). This strongly suggests that formin and profilin work as a module in controlling actin polymerization from the plasma membrane at the tip of pollen tubes.

**FIGURE 2:**
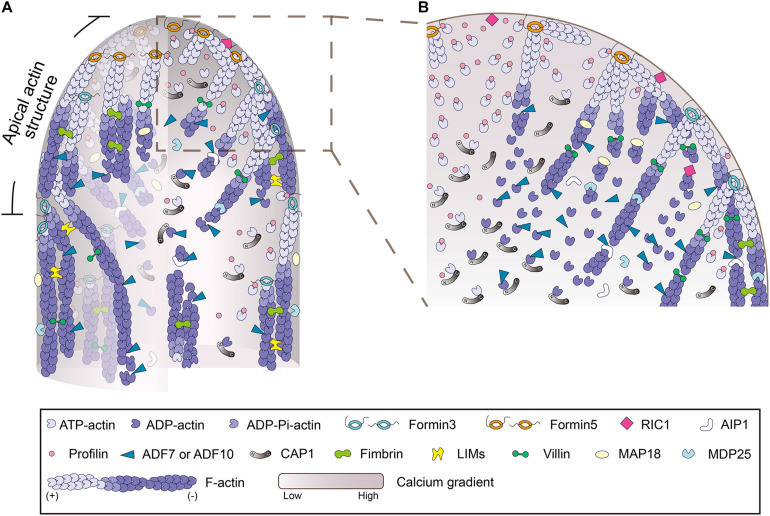
Schematic depiction of the regulation of actin polymerization and dynamics in the pollen tube. **(A)** Schematic depiction of the intracellular localization pattern and function of various ABPs in pollen tube growth domain. The model is mainly based on data from *Arabidopsis*. **(B)** An enlarged picture of the boxed region in **(A)** is presented in order to show more details about the regulation of actin polymerization and dynamics in the tip of pollen tube. In brief, within the pollen tube, actin predominantly exists in the monomeric form. It is buffered by an equimolar amount of profilin to form actin-profilin complexes. Actin polymerization is initiated by membrane-anchored formins, which utilize actin-profilin complexes within the cytoplasm. The membrane-originated actin filaments assume distinct distributions in space as described in Figure 1G, and they are turned over by ADF and its cofactors, including AIP1 and CAP1, and several Ca^2+^-responsive actin severing proteins, which promote the dynamics and control the length of actin filaments. Under the action of various actin bundling/crosslinking proteins, including villins (Qu et al., 2013), LIMs (Wang et al., 2008; Papuga et al., 2010; Zhang et al., 2019), and fimbrins (Su et al., 2012; Zhang et al., 2016), membrane-originated actin filaments are organized into distinct structures and assume distinct distributions in the cortical and inner regions of the pollen tube.

To support continuous actin polymerization during pollen tube growth, a pool of polymerizable actin monomers must be available within pollen tubes. Given that actin is assumed to be buffered by equimolar profilin in pollen, and plant profilins lack or have weak actin nucleotide exchange activity (Kovar et al., 2001; Chaudhry et al., 2007; Liu et al., 2015), a mechanism is required to recharge the dissociated ADP-G-actin before the formation of actin-profilin complexes. Adenylyl cyclase-associated protein 1 (CAP1), also known as Srv2p in budding yeast, is a protein shown to have actin nucleotide exchange activity in *Arabidopsis* (Chaudhry et al., 2007). It is assumed to take on the role of recharging ADP-G-actin in plants. CAP1 is an abundant protein and its cellular concentration falls between that of ADF and profilin in *Arabidopsis* pollen (Jiang et al., 2019). It can coordinate with ADF and profilin to promote actin turnover and enhance actin nucleotide exchange *in vitro* (Chaudhry et al., 2007; Jiang et al., 2019). CAP1 distributes uniformly in pollen tubes and depletion of CAP1 impairs membrane-originated actin polymerization at pollen tube tips. Furthermore, CAP1 protein that is defective in actin nucleotide exchange activity cannot fully rescue the apical actin polymerization defects in *cap1* mutant pollen tubes (Jiang et al., 2019), which suggests that the actin nucleotide exchange activity of CAP1 is biologically significant. These findings together allow us to propose a model for the regulation of actin polymerization from the plasma membrane at pollen tube tips (Figure 2; Jiang et al., 2019). Specifically, the membrane-anchored class I formins initiate actin polymerization from the pool of actin-profilin complexes within the cytoplasm, and ADF drives the turnover of membrane-originated actin filaments and controls their length via its actin severing and depolymerizing activity (see the detailed description below). CAP1 works as the shuttle molecule between ADF and profilin to promote actin turnover and maintain the pool of polymerizable actin monomers to drive formin-mediated actin polymerization from the plasma membrane (Figure 2). These findings provide significant insights into the molecular mechanism that controls actin polymerization from the plasma membrane at the pollen tube tip.

## Regulation of the Turnover and Organization of Membrane-Originated Apical and Subapical Actin Filaments in Pollen Tubes

The mechanisms that regulate the turnover of apical and subapical actin filaments have been the subject of intensive studies in the past. Given that actin filaments are mainly generated from the membrane-anchored class I formins (Cheung et al., 2010; Lan et al., 2018), the ends of actin filaments facing toward the cytoplasm are pointed ends that should be favored by actin-depolymerizing factors (ADFs). ADFs are extremely relevant players in trimming actin filaments to control their length and drive their turnover. Indeed, ADFs have been implicated in the regulation of actin dynamics in pollen grains and pollen tubes (Smertenko et al., 2001; Chen et al., 2002), but the precise mechanism underlying their action remains largely unknown. With the employment of the *Arabidopsis* genetic approach, our understanding of the role and mechanism of action of ADFs in pollen has improved substantially. Besides *Arabidopsis* ADF5, which regulates the actin cytoskeleton via stabilizing and bundling actin filaments in pollen tubes (Zhu et al., 2017), ADF7 and ADF10, which are expressed specifically in *Arabidopsis* pollen (Bou Daher et al., 2011; Daher and Geitmann, 2012), are two major typical actin depolymerizing factors that promote the turnover of the actin cytoskeleton in pollen via severing and depolymerizing actin filaments (Zheng et al., 2013; Jiang et al., 2017). The role of ADF7 in promoting the turnover of shank-localized actin bundles was demonstrated several years ago (Zheng et al., 2013), but its role in regulating the dynamics of apical and subapical actin filaments remains to be characterized. ADF10 was demonstrated to sever and depolymerize subapical actin filaments to promote their turnover and ordering (Jiang et al., 2017). In line with these findings, loss of function of actin-interacting protein 1 (AIP1), the cofactor of ADF (Allwood et al., 2002; Shi et al., 2013; Diao et al., 2020), reduces the rate of actin turnover and induces disorganization of subapical actin filaments in *Arabidopsis* pollen tubes (Diao et al., 2020). In addition, it was shown that depletion of CAP1 decreases ADF-mediated actin depolymerization and severing, which reduces the rate of actin turnover in pollen tubes (Jiang et al., 2019). These data together identify ADF as an essential player in promoting the turnover of actin filaments in pollen tubes.

In addition, as pollen tubes harbor a tip-high Ca^2+^ gradient (HoldawayClarke et al., 1997; Diao et al., 2018), several Ca^2+^-responsive actin severing proteins are involved in regulating the turnover of apical and subapical actin filaments. In this regard, the Ca^2+^-responsive villin/gelsolin/fragmin members are extremely relevant (Yamashiro et al., 2001; Huang et al., 2004; Xiang et al., 2007; Wang et al., 2008; Khurana et al., 2010; Zhang et al., 2010; Zhang et al., 2011; Bao et al., 2012; Wu et al., 2015). Most of the *in vivo* functional data about villin/gelsolin/fragmin family members have come from the analysis of villins using the reverse genetic approach, as the plant genome only encodes genes for full-length villins (Klahre et al., 2000; Huang et al., 2015). The villin homologs were originally identified from pollen by biochemical means and demonstrated to be *bona fide* actin bundlers (Yokota et al., 1998, 2003). Although it was subsequently confirmed that villin can bind to G-actin and promote actin depolymerization in the presence of Ca^2+^/Calmodulin (Yokota et al., 2005), the direct evidence supporting the role of villins in severing actin filaments came from biochemical analyses of villins from *Arabidopsis* and rice (Khurana et al., 2010; Zhang et al., 2010; Zhang et al., 2011; Bao et al., 2012; Wu et al., 2015). In support of the role of villins in promoting actin turnover in pollen tubes, it was shown that loss of function of *Arabidopsis villin2* (*VLN2*) and *VLN5* causes accumulation of filamentous actin at pollen tube tips (Qu et al., 2013). The reduction in the frequency of actin filament severing in *vln2 vln5* double mutant pollen tubes suggests that the severing activity of villins likely contributes to their role in promoting actin turnover (Qu et al., 2013). In line with this finding, it was shown that the severing activity of villin is involved in the formation of actin foci triggered by elevation of the cytosolic Ca^2+^ concentration in pollen tubes (Zhao et al., 2020). Within this framework, several other Ca^2+^-responsive actin severing proteins were also shown to be involved in the regulation of actin turnover at pollen tube tips, such as MAP18, MDP25, and ROP-interactive CRIB motif-containing protein 1 (RIC1) (Zhu et al., 2013; Qin et al., 2014; Zhou et al., 2015). In addition, although there is no evidence showing the direct interaction of RIC3 with the actin cytoskeleton, it was shown that RIC3 promotes the release of free Ca^2+^, which induces actin depolymerization in pollen tubes (Gu et al., 2005). How exactly RIC3 promotes actin turnover in pollen tubes remains to be determined. Nonetheless, these data suggest that the Ca^2+^-responsive actin severing proteins act in concert with the Ca^2+^ gradient to promote actin turnover in pollen tubes.

## The Role of Apical and Subapical Actin Filaments in Regulating Vesicle Traffic in Pollen Tubes

**FIGURE 3:**
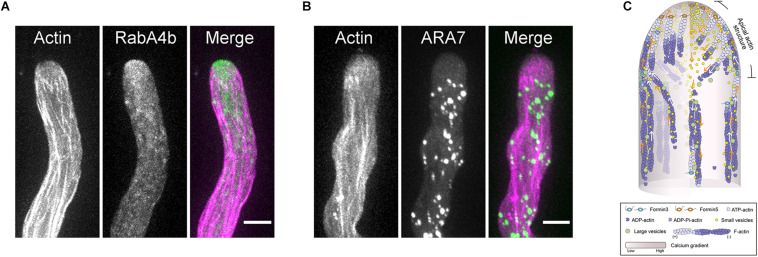
The Role of the Actin Cytoskeleton in Regulating Vesicle Traffic in the Pollen Tube. **(A)** Dual visualization of RabA4b-positive transport vesicles (green) and actin filaments (pink) in a WT *Arabidopsis* pollen tube. The small RabA4b-positive transport vesicles accumulate at the pollen tube tip. Scale bar = 5μm. **(B)** Dual visualization of ARA7-positive endosomes (green) and actin filaments (pink) in a WT *Arabidopsis* pollen tube. The large ARA7-positive endosomes are absent at the pollen tube tip. Scale bar = 5 μm. **(C)** Schematic depiction of the function of the actin cytoskeleton in regulating vesicle traffic in the pollen tube. Both ARA7-positive large endosomes and RabA4b-positive small vesicles are transported along cortical actin bundles in the shank region. Upon reaching the subapex, ARA7-positive large endosomes reverse their direction of movement and return to the base along the inner actin bundles. This is the basis for the generation of reverse fountain cytoplasmic streaming. However, after reaching the subapex, RabA4b-positive small vesicles run straight to the tip along cortical actin bundles within the “apical actin structure,” which leads to the accumulation of vesicles at the pollen tube tip. After reaching the extreme tip, some RabA4b-positive small vesicles will start to move toward the base of the pollen tube. The inner actin filaments within the “apical actin structure” function as physical barrier to prevent their return, which leads to the formation of the “V”-shaped distribution pattern of small vesicles.

It remains largely unknown how actin functions within the apical and subapical regions of pollen tubes. This is partly because we lack a unified view about the organization of actin filaments within that region. As discussed above, one common view is that actin filaments are arrayed into an actin fringe structure at the subapex (Figure 1A; Lovy-Wheeler et al., 2005, 2006). Different hypotheses were raised to explain the function of the actin fringe. The proposed functions include: organizing endomembranes and controlling the location of endo- and exocytotic events; acting as a physical barrier to exclude large organelles; structurally supporting the plasma membrane to facilitate turgor driven extension; and generating the force to drive cell growth (Stephan, 2017). Among the different functions, the actin cytoskeleton plays an obvious role in regulating tip-directed vesicle traffic, which leads to the accumulation of vesicles at the pollen tube tip to support pollen tube growth. Different hypotheses were proposed to explain the role of apical and subapical actin filaments in regulating vesicle traffic in pollen tubes (Geitmann and Emons, 2000). These include spatially constraining the distribution of vesicles (Kroeger et al., 2009), acting as a filter for small vesicles (Kost et al., 1998; Cheung et al., 2008), and acting as the tracks for myosin motors to transport vesicles to the tip (Lovy-Wheeler et al., 2005; Daher and Geitmann, 2011; Chebli et al., 2013). As described above, studies in *Arabidopsis* pollen tubes have revealed more details about the spatial organization and dynamics of the actin cytoskeleton within the apical and subapical regions, which provides an opportunity to understand how actin regulates vesicle traffic in pollen tubes. Within the apical and subapical regions, membrane-originated actin filaments assume distinct spatial distributions, including thick actin bundles in the cortex and relatively fine actin filaments in the middle (Figure 1G; Qu et al., 2017). Further analysis revealed that the cortical actin bundles act as tracks for myosin motors, allowing the transportation of vesicles to the pollen tube tip, while the inner fine actin filaments act as the physical barrier to prevent the backward movement of vesicles from the tip (Qu et al., 2017). This leads to the generation of a “V” shape of vesicle distribution (Figures 3A,C). The apical actin structure as a whole also acts as a physical barrier to prevent the invasion of large organelles into the pollen tube tip (Figures 3B,C). Therefore, cooperation between the apical actin structure and the shank-localized actin bundles leads to the generation of reverse-fountain cytoplasmic streaming and the “V”-shaped vesicle distribution in the pollen tube (Figure 3; Qu et al., 2017). These studies provide significant insights into the functional role of actin in regulating vesicle traffic in pollen tubes.

## Conclusion and Perspectives

Although the essential role of actin in regulating pollen tube growth is well-recognized, the cellular mechanisms underlying the function of actin during pollen tube growth remain to be uncovered. Our understanding of how actin performs its function has been hindered by the lack of a unified view about the origin, spatial organization and dynamics of actin filaments within the growth domain of pollen tubes. Recently, with the introduction of appropriate actin markers and state-of-the-art live cell imaging technologies, along with the usage of mutants lacking different ABPs, our understanding of the origin, polymerization, dynamics, and spatial organization of actin filaments within the growth domain of pollen tubes has improved substantially. Specifically, it is clear that actin filaments are continuously polymerized from the plasma membrane at the extreme apex of pollen tubes during their extension, which answers the long-standing question about whether actin filaments exist at the extreme apex. In addition, actin filaments are polymerized from the plasma membrane at the subapex, where they generate the actin fringe structure reported in pollen tubes (Lovy-Wheeler et al., 2005, 2006). This work provides insights into the origin, polarity and organization of actin filaments within the actin fringe. Together, these findings allow us to conclude that actin filaments within the apical and subapical regions of pollen tubes can be viewed as a whole in terms of their origin, and can be collectively defined as the “apical actin structure” (Qu et al., 2017). Consequently, the pollen tube actin cytoskeleton can be viewed as consisting of two structures: the shank-localized actin bundles and the “apical actin structure” (Figure 1G; Qu et al., 2017). The polymerization of actin filaments from the plasma membrane also occurs in lily and tobacco pollen tubes (Figures 1D,E; Rounds et al., 2014; Qu et al., 2017). This implies that the polymerization of actin filaments from the plasma membrane and formation of the distinct “apical actin structure” might represent a common design for angiosperm pollen tubes.

Careful observations revealed that membrane-originated actin filaments within the pollen tube growth domain assume distinct spatial distributions: they form comparatively thick actin bundles at the cortex and fine actin filaments extending toward the inner region of the cytoplasm (Figure 1G; Qu et al., 2017). The functional coordination of those spatially distinct apical and subapical actin filaments leads to the formation of a “V”-shaped vesicle distribution pattern (Figure 3C; Qu et al., 2017). In addition, the apical actin structure acts as a physical barrier to prevent the apical invasion of large organelles, which facilitates the generation of reverse fountain cytoplasmic streaming (Figure 3C; Qu et al., 2017). However, it remains to be resolved how subapical actin filaments coordinate spatially and functionally with shank-localized actin bundles. Furthermore, given that actin filaments are continuously generated from the plasma membrane at the extreme apex during pollen tube growth, it will definitely be worth exploring how those actin filaments might be involved in the control of exo- and endocytotic events.

As actin polymerization is concurrent with and required for pollen tube growth (Qu et al., 2017), a key area for future research is how growing pollen tubes perceive the upstream signals to control the polymerization and dynamics of actin filaments. Within this framework, another outstanding question is how the activity of membrane-anchored class I formins is precisely regulated. In particular, it will be interesting to investigate how the signaling mediated by ROPs (Li et al., 1999; Gu et al., 2005), phospholipids (Zhang and McCormick, 2010; Zonia, 2010) and the receptor-like kinases (RLKs) (Muschietti and Wengier, 2018) might influence the activity of formins. In particular, as ROPs and RLKs have been implicated in pollen tube guidance (Takeuchi and Higashiyama, 2016; Wang et al., 2016; Luo et al., 2017), it remains to be documented how actin reorganization is involved in the turning of pollen tubes in response to female-derived signals. Establishment of a semi-*in vivo* pollen tube growth system that enables the imaging of actin dynamics at high spatiotemporal resolution might allow us to understand how actin undergoes reorganization during pollen tube turning in response to female-derived attractants. Furthermore, pollen tubes have distinct distributions of ions, such as Ca^2+^ and H^+^ (HoldawayClarke et al., 1997; Messerli and Robinson, 1997; Feijo et al., 1999; Diao et al., 2018), which will influence the activity of ABPs and will in turn impact the dynamics and organization of actin filaments. How actin structures adapt to the cytosolic microenvironment at the pollen tube tip is another interesting question. In summary, plant biologists have made great progress in understanding the dynamics, organization and function of the actin cytoskeleton in pollen tube tips, but many questions still remain to be answered. This promises to be an exciting area of research for many years to come.

## Author Contributions

YX drafted this manuscript. SH conceived this manuscript and revised the writing of this manuscript. Both authors contributed to the article and approved the submitted version.

## Conflict of Interest

The authors declare that the research was conducted in the absence of any commercial or financial relationships that could be construed as a potential conflict of interest.
